# Retrospective Comparison of Empiric Antivenom vs. Expectant Treatment for Eastern Coral Snakebites

**DOI:** 10.5811/westjem.45709

**Published:** 2025-12-20

**Authors:** Reeves Simmons, Chiemela Ubani, Gerard Garvan, Molly Stott, Dawn Sollee, Jay Schauben, Alexandra Derr, Colleen Cowdery, Lindsay Schaack Rothstein, Hayley Gartner, Ashton Federico, Sophia Sheikh

**Affiliations:** * University of Florida Health Jacksonville, Florida/USVI Poison Information Center-Jacksonville, Jacksonville, Florida; † University of Florida College of Medicine-Jacksonville, Department of Emergency Medicine, Jacksonville, Florida; ‡ University of Florida College of Medicine-Jacksonville, Center for Data Solutions, Jacksonville, Florida

## Abstract

**Introduction:**

The coral snake is the only native elapid in North America. Their venom contains potent neurotoxins. Historically, all confirmed/presumed bites were treated with antivenom whether or not symptoms were present. Production of antivenom ceased in 2003. The resultant national shortage prompted clinicians to investigate alternative treatment strategies such as a wait-and-see approach where antivenom is held until signs of systemic toxicity manifest. Now that production has resumed there is limited research available comparing these two treatment paradigms, empiric administration vs the wait-and-see approach. Our objective in this study was to compare outcomes of the two treatment paradigms to determine whether one is associated with better patient outcomes.

**Methods:**

This was a retrospective analysis of coral snakebite cases reported to the Florida Poison Information Center Network from January 1, 1998–December 31, 2021. We collected demographic, clinical, and outcome variables. Patients were stratified into two groups, empiric antivenom administration vs the wait-and-see approach in patients who were asymptomatic in terms of systemic symptoms at the time of initial presentation to the emergency department. We used multivariable logistic regression models, controlling for whether the bite occurred during the North American Coral Snake Antivenin (NACSA) shortage period (yes/no), age, sex, and whether systemic effects developed (yes/no), to determine differences between study groups in the incidence of the main outcomes: intensive care unit (ICU) admission; intubation; and death, as well as ICU and hospital length of stay.

**Results:**

We analyzed 301 cases: 171 (56.8%) empiric; and 130 (43.2%) wait-and-see. Patients in the empiric treatment group had approximately three times higher likelihood of ICU admission (empiric 121 [75.2%] and wait-and-see 71 [56.8%]), odds ratio [OR} 3.047, P = .05). There was no difference in the incidence of intubation (empiric 2 [1.2%] and wait-and-see 1 [<1%]), OR 2.486, P = .63) or in ICU length of stay (OR 0.485, P = .08). Of the patients treated with NACSA (191), adverse reactions to the antivenom occurred in 38 (19.9%) patients—35 patients in the empiric group and three in the wait-and-see group who later received antivenom. Of these 38 patients, eight (21.1%) experienced an anaphylactic reaction.

**Conclusion:**

Empiric North American Coral Snake Antivenin administration was associated with higher ICU admissions and with a considerably higher risk of adverse reactions, which may serve to impose caution when treating empirically.

## INTRODUCTION

In North America there are three species of native coral snakes. However, only two species, *Micrurus fulvius* (Eastern coral snake) and *Micrurus tener* (Texas coral snake) produce highly potent venom and, therefore, are of high clinical concern. Their venom contains potent neurotoxins that bind the muscarinic acetylcholine receptors at the neuromuscular junction and may lead to neurologic symptoms such as paresthesias, diplopia, ptosis, difficulty swallowing, muscle weakness, and respiratory paralysis. Symptoms may be delayed as late as 13 hours post envenomation.^1^ Only one documented death has been reported in the literature due to an envenomation from an Eastern coral snake. Of note, this patient failed to seek medical attention/treatment after the bite.^2^

The package insert for Pfizer’s North American Coral Snake Antivenin (NACSA) recommends it not be administered prophylactically to asymptomatic patients (listed under contraindications section of package insert).^3^ Historically, all patients with a presumed or documented coral snake envenomation were recommended to receive empiric NACSA treatment regardless of symptoms.^4^ The reason for this recommendation likely stems from NACSA’s inability to reverse neurologic symptoms once present. However, NACSA may limit further progression of these symptoms, potentially preventing the need for mechanical ventilation in cases where respiratory muscle weakness has not yet developed.

An unintentional consequence of the empiric approach is the administration of antivenom in cases of “dry bites,” which occur when no venom is introduced into the patient after a bite. The rate of dry bites is unknown but may be as high as 50%.^5^ Additionally, published rates for adverse reactions to NACSA administration are quite high (up to 18%).^4^ Unlike North American crotalid antivenoms, which are Fab or F(ab’)_2_ fragments, NACSA is a whole immunoglobulin G antibody, likely contributing to its high immunogenicity rate. Therefore, empiric NASCA administration in a person who may not have been envenomated, may potentially result in unnecessary treatment and risk development of a significant adverse drug reaction.

The production of NACSA was discontinued in 2003 leading to a national shortage of the only approved coral snake antivenom approved by the US Food and Drug Administration (FDA), prompting the FDA to extend the expiration date of certain NACSA lot numbers.^6^ During this shortage, the treatment paradigm shifted, and many experts adopted a “wait-and-see” (WaS) approach, recommending NACSA at the first sign of systemic toxicity, rather than empirically treating all confirmed/suspected bites. In recent years, the availability of NACSA has increased due to the resumption of product manufacturing. Research is limited comparing the two treatment paradigms (empiric vs WaS) to determine whether one approach is associated with better patient outcomes and reduced healthcare costs. Specifically, it is unknown whether delaying NACSA treatment until onset of systemic symptoms leads to increased morbidity, extended intensive care unit (ICU) stays, or prolonged mechanical ventilation. Existing research, albeit limited, suggests similar patient outcomes with either approach.^4^

**Population Health Research Capsule**

What do we already know about this issue?

*Coral snakebites can cause severe neuromuscular symptoms; however, North American Coral Snake Antivenin (NACSA) use carries a high rate of adverse effects*.

What was the research question?

*Was a wait-and-see approach associated with improved outcomes compared to empiric antivenom in asymptomatic coral snakebite patients?*

What was the major finding of the study?

*Empiric vs wait-and-see adverse reactions were 20.5% vs 2.3%, OR 1.46, 95% CI 0.405–5.258), and ICU admission 75.2% vs 56.8%, OR 3.05, 95% CI 1.02–9.57, P = .05*.

How does this improve population health?

*Findings support cautious antivenom use in asymptomatic bites to reduce risk of adverse drug reactions, andemphasizes importance of ICU monitoring for delayed neuromuscular symptoms*.

Our primary objective was to compare the incidence of intubation, hospital and ICU length of stay (LOS), and National Poison Data System (NPDS) outcome code (death, major, moderate, minor, no effect) between the empiric and WaS treatment groups.^7^

## METHODS

### Study Design and Population

This was a retrospective observational cohort study of cases managed by the Florida Poison Information Center Network (FPICN) and was approved by the University of Florida Institutional Review Board. The FPICN consists of three poison centers, located in Jacksonville, Miami, and Tampa, which assess, triage, and provide management recommendations to healthcare facilities (HCF) and the public in the state of Florida and the US Virgin Islands. A unique patient chart is created for each exposure call made to the FPICN and is stored in the FPICN’s electronic medical records database. This database includes nationally standardized coded data elements, following codes provided by America’s Poison Centers’ NPDS, as well as non-coded or free-text fields (ie, notes) where additional information related to the exposure can be documented. Additional non-NPDS data fields added to the FPICN’s system unilaterally enhance data surveillance and patient management capabilities.^7^

We completed a query of the FPICN database for human coral snake exposures occurring between January 1, 1998–December 31, 2021 using NPDS generic codes related to “coral snake” exposures. Cases were excluded if 1) the patient was symptomatic from the bite (other than pain and allergic reaction to snake venom) at the time of presentation; 2) the exposure did not involve an actual bite; 3) the patient was monitored for less than 24 hours post exposure at a HCF; or 4) the NPDS medical outcome code was “not followed,” “unable to follow,” or “exposure probably not responsible for effect.” We followed the guidelines described by Worster et al for elements that should be included in studies involving data collected from medical charts.^8^ All elements were performed, except that abstractors were not blinded to the study objectives and variables with missing data were excluded from data analysis.

### Variables Collected

After defining variables to be collected and receiving training, four independent non-blinded reviewers (MS, AD, CC, AF) extracted data from the FPICN database using a standardized data collection tool. A fifth reviewer (CU) then performed a secondary review and with a sixth reviewer (SS) resolved any conflicting coding. Inter-rater reliability was 99%, with only 10 instances requiring resolution.

### Demographics and Bite Characteristics

Collected demographics and characteristics of the bite included age, sex, date of bite, whether the snake matched the physical description of an Eastern coral snake (black-headed, with red and yellow contiguous bands) (ie, positive coral snake identification), whether the snake had to be forcibly removed (a classic feature characteristic of coral snakebites),^9^ anatomical location of the bite, whether the bite broke skin, presence of scratches or abrasions, redness, or edema around the bite site, and whether the bite occurred during the time period of NACSA shortage 2003–2019 (as determined after email correspondence with Pfizer representative).

### Clinical Effects

The following NPDS clinical effects were collected: decreased level of consciousness; ptosis; diplopia; blurred vision; nausea; emesis; dysphagia; dysphonia; numbness or paresthesias; fasciculations; weakness; seizures; paralysis; respiratory depression; pain; any allergic reactions to the snake venom itself, and whether a death occurred. Systemic clinical effects were defined as having any of the following: decreased level of consciousness; ptosis; diplopia; blurred vision; nausea; emesis; dysphagia; dysphonia; numbness or paresthesias; fasciculations; muscular weakness; seizures; paralysis; and respiratory depression.

### Adverse Effects of North America Coral Snake Antivenin

Adverse effects related to NACSA administration included anaphylaxis, angioedema, pruritus, dermal reactions (hives, rash, and/or welts), shortness of breath, and hypotension (defined as systolic blood pressure < 100 mm Hg or diastolic blood pressure < 60 mm Hg). Treatments and interventions provided for NACSA adverse effects were also collected, including intubation, epinephrine, antihistamines, and corticosteroids.

### Clinical Outcomes

Clinical outcome data collected included hospital and ICU admission (yes/no), LOS (days), intubation for respiratory failure from snakebite (yes/no), and NPDS medical outcomes (no effect, minor effect, moderate effect, major effect, death). See Supplemental Materials for NPDS outcome definitions.^7^

### Data Analysis

Patients were stratified into two groups (empiric vs WaS) based on NACSA administration in patients who were asymptomatic (in terms of systemic effects) at the time of initial presentation to the emergency department. (ED). Patients in the “empiric” group were those who received NACSA and were asymptomatic. Patients in the WaS group did not receive antivenom upon initial presentation and were also asymptomatic. Patients who had symptoms at the time of presentation were excluded from this analysis. Since group assignment was designated based on NACSA administration at the time of ED presentation in patients who were asymptomatic, it was possible for patients who were initially asymptomatic (thus meeting study inclusion) to later manifest symptoms necessitating antivenom treatment. These patients were retained in the study as our objective was to determine best approaches for NACSA administration in patients who are asymptomatic at the time of ED presentation.

Logistic regressions were run to determine differences in demographics (age, sex, whether bite occurred during NACSA shortage year [yes/no]) and snakebite [positive identification of coral snake [yes/no] and snake forcibly removed (yes/no)] between the empiric and WaS groups.

### Multivariable Regressions

We used multivariable logistic regression models, controlling for whether the bite occurred during the NACSA shortage period (yes/no), age, sex, and whether systemic effects developed (yes/no), to determine differences between study groups in the incidence of ICU admission, intubation, and death, as well as ICU and hospital LOS. The ICU and hospital LOS were modeled using a multivariable zero-inflated negative binomial regression. Missing or unknown data were dropped from the analysis. We performed all statistical analyses in R 4.1 (The R Foundation for Statistical Computing, Vienna, Austria).

## RESULTS

### Demographics and Snakebite Characteristics

**Figure 1:**
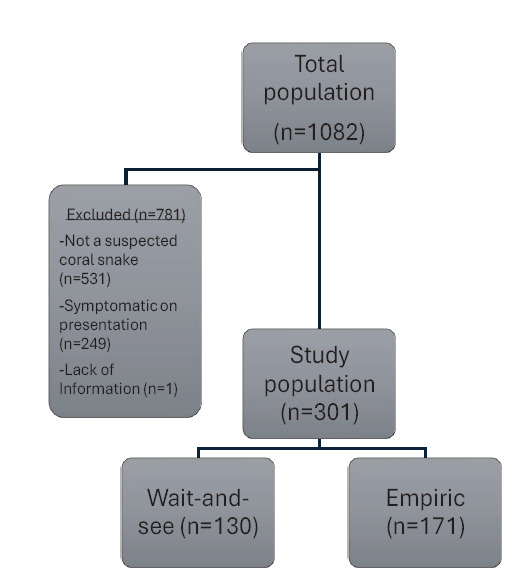
Patient screening and inclusion for empiric antivenom administration vs wait-and-see approach in cases of coral snake envenomation.

We identified 1,082 cases based on the search criteria. Of these cases, 781 were removed based on exclusion criteria, leaving 301 cases for analysis. Of the 301 cases, 171 were grouped into the empiric group and 130 in the WaS group (Figure 1).

**Figure 2:**
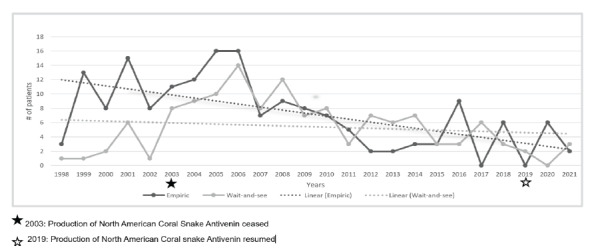
Demographics and snakebite characteristics for empiric antivenom administration vs wait-and-see treatment approach for coral snake exposures managed by the Florida Poison Information Center Network.

Twenty (15.4%) patients in the WaS group were later administered NACSA after development of systemic effects. Figure 2 depicts the number of patients in each group per year over the study period. Prior to 2006 the empiric treatment paradigm was the more common approach. Starting in 2007, however, more patients were managed using the WaS approach with a peak in 2008. Over the study period, the empiric treatment approach declined from 2008 to 2012. The predominance of one treatment paradigm over the other varied year to year since the initial cessation of NACSA production and continued to fluctuate even after production resumed.

Table 1 provides demographics and bite characteristics for each group. Mean age (*P* =.93) and sex distribution (*P* = .58) were similar between groups. Most of the patients managed using the WaS approach presented during the NACSA shortage period (87.7%, *P* < .01). Positive coral snake identification (*P* = .01) and forcible removal of the snake (*P* < .01) were more common in the empiric group than the WaS group.

### Clinical Effects and Treatments

Table 2 compares clinical effects from the bite between groups. Most patients did not develop systemic effects (empiric [103, 60.2%]; WaS [94, 72.3%]). No patients developed seizures, fasciculations, or diplopia. No bites resulted in death. One report each of dysphonia and paralysis were noted in the empiric group. One patient in the WaS group developed respiratory depression and was subsequently intubated.

### Adverse Effects. Of of North America Coral Snake Antivenin

Table 3 compares NACSA adverse effects and subsequent interventions between groups that were reported to the FPICN. A total of 191 patients received antivenom. All patients in the empiric group (171) received NACSA. and 20 (15.4%) patients in the WaS group (130) received NACSA later in their clinical course once systemic symptoms developed. (See Table 2 for characterization of symptoms.) Of the 191 patients treated with NACSA), 38 (19.9%) developed an adverse reaction to the antivenom itself (35 in the empiric and three in the WaS group), and eight (21.1%) of these 38 patients had an anaphylactic reaction. Overall, the most common adverse reactions reported were hives/rash/welts (12%), itching (12%), and shortness of breath (5.2%). In the empiric group 68 adverse effects to the NACSA were reported in 35 (20.5%) individual patients. A total of five adverse effects to NACSA were reported in three individual patients within the WaS group. Within the empiric group two patients required intubation secondary to adverse effects from NACSA.

### Multivariable Logistic Regression Models

Table 4 displays the results of the regression analysis. The empiric group was more likely to be admitted to the ICU (*P* = .05) and hospital (*P* = .03) compared to the WaS group. However, there were no statistical differences when looking at the incidence of intubation, average ICU LOS, average hospital LOS, and NPDS medical outcome codes.

## DISCUSSION

In this study, we compared two treatment approaches, empiric NACSA administration vs the WaS approach, for suspected coral snake envenomation to determine temporal trends and differences in patient outcomes and hospital resource utilization. We found the prevalence of the two approaches differed over time. The incidence of hospital and ICU admissions differed between groups. There were no differences between groups in incidence of intubation, ICU or hospital LOS, or in NPDS medical outcomes. Additionally, we found relatively high rates of adverse effects after NACSA administration. Based on our study population, outcomes after a potential coral snakebite are overall generally positive, with no deaths reported and relatively few cases designated as having a major NPDS medical outcome.

Use of the two treatment approaches varied over the study period. The production of NACSA ceased in 2003. Until that time, the empiric approach was more common and remained so even during the initial years after production of NACSA ceased, likely due to hospitals having a reserve of NACSA in stock. However, in 2007, for the first time during our study period, the WaS approach became more common, peaking in 2008. After 2008, use of each treatment approach fluctuated year to year, likely due to a multitude of factors, including extension of NACSA lot-expiration dates by the FDA, changes in management practices fostered during the NACSA shortage, relatively few NACSA administrations per year, and antivenom procurement issues.

We were curious to see whether certain snakebite characteristics may contribute to a clinician’s decision to empirically treat. Positive coral snake identification and forcible removal of the snake were characteristics associated with the empiric treatment approach. Forcible removal of the biting snake, with little to no localized symptoms or evidence of bite marks, is a unique characteristic of coral snakebites as they have short, fixed front fangs, and often latch onto victims and deliver venom through a chewing action. (Note: chewing is not required to deliver venom.)^10^

Notably, pediatric patients were less likely to receive empiric treatment. Several factors may explain this, including higher proportion of pediatric cases occurring during the antivenom shortage, limited information about the snake encounter, limited access to antivenom, or heightened clinician concern regarding the risk of serious allergic reactions. Further studies are needed to determine treatment considerations/patterns for pediatric populations with coral snake envenomations.

Interestingly, despite the increased likelihood of hospital and ICU admissions in the empiric group, we found no difference between groups in either hospital or ICU LOS, intubation incidence, or NPDS outcomes, suggesting that both groups had similar clinical severity and that receipt of NACSA alone may have been the driving factor for ICU admission. This finding is concerning. The FPICN recommendation after a suspected coral snakebite is to perform hourly neurological examinations for at least 24 hours, *regardless* of patient symptoms (whether present or absent) or administration of antivenom. This frequency of assessment is generally only feasible in an ICU setting, and its recommendation based upon data demonstrating the potential development of delayed neurological effects.^1^ Thus, there should not have been a difference in the number of ICU admissions or hospital admissions between the groups. Potential factors contributing to this difference could have been ED crowding, prolonged boarding times, or clinician underestimation of the potential morbidity associated with coral snake envenomations. Patients not monitored in an ICU may be at risk for delayed identification of systemic symptoms from the venom or allergic reactions from the antivenom, possibly leading to adverse outcomes.

In our cohort, a relatively low incidence (5%) of serious neuromuscular symptoms (such as ptosis, blurred vision, dysphagia, and muscular weakness) developed after envenomation. However, a relatively high percentage of patients (~20%) developed an adverse reaction to the NACSA administration. Currently the package insert advises not to provide NACSA to asymptomatic patients.^3^ This recommendation is likely due to the high rate of adverse effects previously noted in the literature, which was again demonstrated in this study.^4^ The incidence of intubation in this cohort was too low for accurate comparisons between groups; however, it should be noted that two of the three patients requiring intubation in our cohort, were intubated due to adverse reactions attributed to NACSA administration rather than the neurotoxic effects of the venom itself. Given these results collectively, the likelihood of developing systemic symptoms needs to be weighed against the possible risks associated with empiric NACSA administration, provided that patients are monitored in an ICU setting for the recommended time. Additional studies are needed to further understand the risk-benefit profile of empiric antivenom vs a wait-and-see approach when managing coral snake envenomations.

Poison information centers or local experts should emphasize to treating clinicians the risk of adverse effects that may result due to administration of NACSA and/or the possibility of delayed neurological symptoms due to the envenomation, along with the importance of proper monitoring and duration of evaluation for all patients after a coral snakebite. Regardless of the treatment strategy, all patients with a coral snake envenomation should be transported to a facility that has NACSA or that can procure sufficient NACSA.

## LIMITATIONS

There are several limitations that should be noted. First, this study was a retrospective chart review of cases reported to the FPICN. As with all poison center data, data are reported by the caller/HCF and are subject to recall bias and reporting of limited or incomplete data, depending on what information is available at the time of the initial call and during follow-up calls made by the poison center staff. Second, all potential NACSA adverse effects that may have developed days to weeks after hospital discharge, such as serum sickness, could not be reliably reported secondary to limited patient follow-up. Thus, the incidence may be higher than what is reported in this paper. Third, there was inconsistent monitoring frequency by HCFs. The FPICN recommendations are to monitor for 24 hours using hourly neurological examinations. However, due to staffing, bed availability, and other factors, some HCFs did not follow these recommendations; therefore, it is possible that serious symptoms did manifest but were not identified or reported by the HCF. Fourth, it is notable that a higher percentage of patients in the empiric treatment group developed systemic effects, despite the fact that the primary goal of antivenom is to prevent primary development/progression of systemic toxicity. This apparent discrepancy may reflect a tendency to empirically treat patients with clinical features suggestive of more severe envenomation—such as bites that broke the skin and forcible snake removal—both of which were more common in the empiric group. Alternatively, subtle early signs of systemic toxicity may have been under-recognized in the WaS group, potentially contributing to differences in outcomes.

Fifth, we were unable to determine how many patients from our cohort were envenomated and how many received a “dry” bite (a bite where little to no venom is released). Sixth, there is ambiguity regarding the exact timing of when NACSA production ceased and when it resumed. We used estimated time frames based on our knowledge of the NACSA supply within our network’s treatment area and FDA notices, as well as communications with Pfizer. Additionally, our analysis focused on the incidence of systemic symptoms; therefore, we did not evaluate the presence of localized symptoms other than pain and edema. However, we do not feel this limitation impacts our findings as localized symptoms after a coral snakebite are not common and the focus of this study was examining clinical symptoms used to determine indications for NACSA administration.

## CONCLUSION

Empiric administration of North American Coral Snake Antivenin was associated with higher ICU admissions. However, there was no difference in ICU length of stay, intubation incidence, or clinical outcome measures. Given the relatively high risk of adverse effects from NACSA administration and low incidence of neuromuscular symptoms following envenomation, clinicians should exercise caution when considering empiric treatment.

## Supplementary Information



## Figures and Tables

**Table 1:** Demographics and snakebite characteristics for empiric antivenom administration vs wait-and-see treatment approach for coral snake exposures managed by the Florida Poison Information Center Network.

Demographics	Empiric (171, 56.8%)	Wait-and-see (130, 43.2%)	Overall (301, 100%)
Age*	35 [23,46]	37 [18,49]	37 [20,47]
Female	20 (11.7)	19 (14.6)	39 (13)
Male	151 (88.3)	111 (85.4)	262 (87)
Pediatric (<18 years)	28 (16.4)	30 (23.1)	58 (19.3)
Shortage year			
Yes	116 (67.8)	114 (87.7)	230 (76.4)
No	55 (32.2)	16 (12.3)	71 (23.6)
Snakebite characteristics			
Positive snake Identification/ description			
Yes	144 (84.2)	108 (83.1)	252 (83.7)
No	6 (3.5)	15 (11.5)	21 (7)
Unknown	21 (12.3)	7 (5.4)	28 (9.3)
Snake forcibly removed			
Yes	60 (35.1)	27 (20.8)	87 (28.9)
No	44 (25.7)	70 (53.8)	114 (37.9)
Unknown	67 (39.2)	33 (25.4)	100 (33.2)
Bite broke skin			
Yes	118 (69)	66 (50.8)	184 (61.1)
No	19 (11.1)	45 (34.6)	64 (21.3)
Unknown	34 (19.9)	19 (14.6)	53 (17.6)
Fang marks/puncture wound			
Yes	86 (50.3)	56 (43.1)	142 (47.2)
No	36 (21.1)	55 (42.3)	91 (30.2)
Unknown	49 (28.7)	19 (14.6)	68 (22.6)
General area of bite			
Finger	92 (53.8)	75 (57.7)	167 (55.5)
Hand/Arm	50 (29.2)	26 (20)	76 (25.3)
Leg/foot/toe	12 (7.1)	17 (13.1)	29 (9.6)
Other/unknown	17 (9.9)	12 (9.2)	29 (9.6)
Edema			
Yes	62 (36.3)	27 (20.8)	89 (29.6)
No	63 (36.8)	91 (70)	154 (51.2)
Unknown	46 (26.9)	12 (9.2)	58 (19.3)

* Median (interquartile range).

**Table 2:** Clinical effects in empiric antivenom administration vs wait-and-see treatment approach for coral snake exposures managed by the Florida Poison Information Center Network.

Systemic clinical effects	Empiric (171, 56.8%)	Wait-and-see (130, 43.2%)	Overall (301, 100%)
Developed systemic effects			
Yes	68 (39.8)	36 (27.7)	104 (34.6)
No	103 (60.2)	94 (72.3)	197 (65.4)
Decreased level of consciousness			
Yes	1 (<1)	1 (<1)	2 (<1)
No	170 (99.4)	129 (99.2)	299 (99.3)
Ptosis			
Yes	1 (<1)	2 (1.5)	3 (1.0)
No	170 (99.4)	128 (98.5)	298 (99.0)
Blurred vision			
Yes	1 (<1)	3 (2.3)	4 (1.3)
No	170 (99.4)	127 (97.7)	297 (98.7)
Emesis			
Yes	5 (2.9)	4 (3.1)	9 (3.0)
No	166 (97.1)	126 (96.9)	292 (97.0)
Dysphagia			
Yes	2 (1.2)	0 (0.0)	2 (<1)
No	169 (98.8)	130 (100)	299 (99.3)
Paresthesia			
Yes	21 (12.3)	18 (13.8)	39 (13)
No	150 (87.7)	112 (86.2)	262 (87)
Muscular weakness			
Yes	3 (1.8)	3 (2.3)	6 (2.0)
No	168 (98.2)	127 (97.7)	295 (98.0)
Nausea			
Yes	6 (3.5)	3 (2.3)	9 (3.0)
No	165 (96.5)	127 (97.7)	292 (97.0)
Allergic reaction to snake venom			
Yes	3 (1.8)	1 (<1)	4 (1.3)
No	168 (98.2)	129 (99.2)	297 (98.7)

**Table 3:** Antivenom adverse effects and interventions in empiric antivenom administration vs wait-and-see treatment approach for coral snake exposures managed by the Florida Poison Information Center Network.

	Empiric (171, 89.5%)	Wait-and-see (20, 10.5%)	Overall (191, 100%)
Antivenom Adverse Effects (AAE)			
Anaphylaxis from antivenom			
Yes	8 (4.7)	0 (<1)	8 (4.2)
No	163 (95.3)	20 (100)	183 (95.8)
Systolic < 100 mm Hg			
Yes	2 (1.2)	0 (<1)	2 (1)
No	166 (97)	20 (100)	186 (97.4)
Unknown	3 (1.8)	0 (<1)	3 (1.6)
Diastolic < 60 mm Hg			
Yes	4 (2.3)	0 (<1)	4 (2.1)
No	164 (95.9)	20 (100)	184 (96.3)
Unknown	3 (1.8)	0 (<1)	3 (1.6)
Angioedema			
Yes	3 (1.8)	0 (<1)	3 (1.5)
No	168 (98.2)	20 (100)	188 (98.5)
Itching			
Yes	20 (11.7)	3 (15)	23 (12.0)
No	151 (88.3)	17 (85)	168 (92.4)
Hives, rash, welts			
Yes	21 (12.3)	2 (10)	23 (12.0)
No	150 (87.7)	18 (90)	168 (88.0)
Shortness of breath			
Yes	10 (5.8)	0 (<1)	10 (5.2)
No	161 (94.2)	20 (100)	181 (94.8)
Interventions for AAE			
Intubation			
Yes	2 (1.2)	0 (<1)	2 (1)
No	169 (98.8)	20 (95)	189 (99)
Epinephrine			
Yes	8 (4.7)	0 (<1)	8 (2.7)
No	163 (95.3)	19 (95)	182 (95.3)
Unknown	0 (<1)	1 (5)	1 (<1)
Antihistamines			
Yes	32 (18.7)	3 (15)	35 (18.3)
No	137 (80.1)	16 (80)	153 (80.1)
Unknown	2 (1.2)	1 (5)	3 (1.6)
Steroids			
Yes	25 (14.6)	3 (15)	28 (14.7)
No	144 (84.2)	16 (80)	160 (83.8)
Unknown	2 (1.2)	1 (5)	3 (1.6)

**Table 4:** Multivariable logistic regression models comparing study outcomes between empiric antivenom administration and wait-and-see treatment approach.

	Empiric (171, 56.8%)	Wait-and-See (130, 43.1%)	OR (95% CI)	P-value
Hospital admission			4.536 (1.206, 19.890)	.03
Yes	170 (99.4)	118		
No	1 (0.6)	12		
Hospital length of stay*			1.517 (0.831, 2.911)	.19
Days	1 [1,2]	1 [1,2]		
Admitted to ICU			3.047 (1.024, 9.573)	.05
Yes	121 (75.2)	71 (56.8)		
No	40 (24.8)	54 (43.2)		
Unknown	10	5		
ICU length of stay (days)			0.485 (0.210, 1.058)	.08
0	4 (3.3)	0 (<1)		
1	83 (68.6)	54 (76.1)		
2	27 (22.3)	14 (19.7)		
3	4 (3.3)	2 (1.5)		
≥ 4	2 (1.7)	1 (1.4)		
Unknown	1 (<1)	0 (<1)		
Intubation			2.486 (0.071, 171.253)	.63
Yes	2 (1.2)	1 (<1)		
No	169 (98.8)	129 (99.2)		
NPDS outcomes code			1.475 (0.935, 2.369)	.10
Major effect	7 (4.1)	2 (1.5)		
Moderate effect	33 (19.3)	23 (17.7)		
Minor effect	123 (71.9)	82 (63.1)		
No effect	8 (4.7)	23 (17.7)		

* Median (interquartile range).

*ICU*, intensive care unit; *OR*, odds ratio; *NPDS*, National Poison Data System.
