# Supplementary material for: Retrospective Comparison of Empiric Antivenom vs. Expectant Treatment for Eastern Coral Snakebites
Source: West J Emerg Med. 2025 Dec 20;27(1):167–76. doi: 10.5811/westjem.45709 (PMC12815500; doi:10.5811/westjem.45709)
Supplement: Supplementary file 1 [file wjem-27-167-s001.docx]

**Appendix A: National Poison Data System (NPDS) Outcome Code Definitions** ^7^

- **No effect:** the patient developed no symptoms as a result of the exposure.
- **Minor Effect:** the patient exhibits some symptoms as a result of the exposure, but they were minimally bothersome to the patient. The patient has returned to a pre-exposure state of well-being and has no residual disability or disfigurement.
- **Moderate Effect:** The patient exhibited symptoms as a result of the exposure which are more pronounced or more of a systemic nature than minor symptoms. Usually some form of treatment is or would have been indicated.
- **Major:** The patient has exhibited symptoms as a result of the exposure which were life-threatening or resulted in significant residual disability or disfigurement.
- **Death:** The patient died as a result of the exposure or as a direct complication of the exposure where the complication was unlikely to have occurred had the toxic exposure not preceded the complication.
